# Effect of drought on root exudates from *Quercus petraea* and enzymatic activity of soil

**DOI:** 10.1038/s41598-022-11754-z

**Published:** 2022-05-10

**Authors:** Karolina Staszel, Jarosław Lasota, Ewa Błońska

**Affiliations:** Department of Ecology and Silviculture, Faculty of Forestry, University of Agriculture in Krakow, 29 Listopada 46 Str., 31-425 Kraków, Poland

**Keywords:** Forestry, Climate-change mitigation

## Abstract

Root exudation is a key process that determines rhizosphere functions and plant-soil relationships. The present study was conducted with the objectives to (1) determine the root morphology of sessile oak seedlings in relation to drought, (2) assess root exudation and its response to drought, and (3) detect possible changes in the activity of soil enzymes in response to drought enhancement. In the experiment, sessile oak seedlings (*Quercus petraea* Matt.) were used, and two variants of substrate moisture (25% humidity—dry variant and 55% humidity—fresh variant) on which oaks grew were considered. Exudates were collected using a culture-based cuvette system. Results confirmed the importance of drought in shaping the morphology of roots and root carbon exudation of sessile oak. The oak roots in the dry variant responded with a higher increment in length. In the case of roots growing in higher humidity, a higher specific root area and specific root length were determined. Experimental evidence has demonstrated decreased root exudation under dry conditions, which can lead to a change in enzyme activity. In the study, enzyme activity decreased by 90% for β-D-cellobiosidase (CB), 50% for β-glucosidase (BG) and N-acetyl-β-D-glucosaminidase (NAG), 20% for β-xylosidase (XYL) decreased by, and the activity of arylsulphatase (SP) and phosphatase (PH) decreased by 10%.

## Introduction

The conducting observations and measurements of climate elements confirm that the climate changes on a global scale, with the tendency of air temperature increase. The global average temperature is projected to increase between 1.5 °C and 6.4 °C by 2100^1^. An undoubted effect of changes in thermal conditions, wind speed, and amount of precipitation will be the occurrence of extreme meteorological phenomena, including rapid and strong periods of drought^2^. Such an increase air temperatures and simultaneous changes in rainfall distribution during the year affect plant growth and, among others, root systems. Früchtenich et al.^3^ indicate that mesic, deciduous European oaks (*Q. robur* and *Q. petraea*) tend to use an avoidance strategy to cope with potential drought stress on sandy soil by establishing deep root systems, thereby increasing access to water supply during the initial growth phase. Root development may be affected directly by elevated soil temperatures or indirectly by changes in the physiology, development, and resource acquisition of the shoot in response to warmer air temperatures or by a combination of both factors^4^. An earlier study had also demonstrated that root exudates are active players in whole-ecosystem responses to environmental perturbation^5^. Furthermore, by collecting root exudates from intact root systems in solution, many studies have shown that drought affects the composition of root exudates^6,7^. Root exudation is a crucial process that determines rhizosphere functions and plant-soil relationships^8^. Root exudation is a key plant function with a large influence on soil organic matter dynamics and plant-soil feedbacks in forest ecosystems^9^.

Plant exudates also play an essential role in the shaping of soil extracellular enzyme activity. They may have a significant effect on the expression and repression of extracellular enzyme activity in the rhizosphere. According to Gianfreda^10^, the enzyme activity profile of the rhizosphere is a footprint of plant-microorganism interactions. Soil enzymes, as indicators of microbial nutrient demand and metabolic processes, play an important role in soil organic carbon (C) mineralization, the nitrogen (N) cycle, the phosphorus (P) cycle, and the sulfur (S) cycle^11^. Soil enzymes are central in the response of terrestrial ecosystems to climate change, and studying these enzymes can be crucial for the models’ implementation^12^. Under drought conditions, enzyme production and activity are reduced as the nutrient requirement for enzyme production exceeds the net increase in nutrient availability for microbes^13^. For example, Sardans and Penuelas^14^ found that the enzymes involved in the N cycle, protease and urease, were affected the most by drought, and the reduction of soil moisture decreased enzyme activity.

The study covered sessile oak, which is the basic forest-forming species in a temperate climate in Europe, spcifically. Despite the fact that it is a species with a relatively high tolerance to lower soil moisture, simulations of changes in climatic conditions together with an assessment of the species' reactions suggest the possibility of a significant reduction in its survival and deterioration of growth conditions in the areas of its south-eastern range^15,16^. This research is an attempt to improve the understanding of how sessile oak trees respond to drought in temperate climate. The present study was conducted with the aims to (1) determine root morphology of sessile oak seedlings (*Quercus petraea* L.) in relation to drought, (2) assess root exudation and its response to drought, and (3) detect the possible changes in the activity of soil enzymes in response to drought enhancement.

## Materials and methods

### Description of experiment

The experiment was carried out in the Laboratory of Forest Environment Geochemistry and Land Intended for Reclamation at the Faculty of Forestry at the University of Agriculture in Krakow. In the experiment, 3-month-old sessile oak seedlings were used. Oaks grew in a typical substrate used in forest nurseries, the properties of which are presented in Table 1. The growth condition of sessile oak seedlings was assessed in line with Regulation of the Minister of the Environment^17^. Seedlings with similar growth characteristics were selected for experiment. The single seedlings were grown in uniform plastic pots 15 cm in diameter and height. The research considered two variants of substrate moisture (25% humidity – dry variant and 55% humidity – fresh variant) on which oaks grew. The humidity sensors in an hourly interval were used to monitor the soil moisture in the post. In the event of change in soil humidity it was brought back to the initial state. The experiment was conducted from March 20 to April 20, 2021. Samples for analysis were taken four times every week. In each variant of moisture and each campaigns of the experiment four seedlings were samples from one pots. The completed experiment complies with local and national regulations.

**Table 1:** The physico-chemical characteristics of substrate used for experiment.

pH H_2_O	pH KCl	N	C	C/N	Hh	Hex	P	Ca	K	Mg	Na
3.99	2.81	0.70	48.82	70.0	62.21	4.00	89.60	3.53	1.08	1.45	0.01

C, N (%);Hh– hydrolytic acidity (cmol( +)·kg^-1^), Hex—exchangeable acidity (cmol( +)·kg^-1^); P, Ca, K, Mg and Na (cmol( +)·kg^−1^).

### Root exudate collection

Exudates were collected in four sampling campaigns during the experiment using a culture-based cuvette system^18^. In each variant of humidity, four oaks were sampled. Root exudates were collected from one branched fine root segments of similar length and branching. Each root system was carefully removed with deionized water and fine forceps to maintain the integrity of the root. The root systems were placed into a sterile glass syringe with sterile glass beads moistened with a C-free nutrient solution (0.5 mM NH_4_NO_3_, 0.1 mM KH_2_PO_4_, 0.2 mM K_2_SO_4_, 0.15 mM MgSO_4_, 0.3 mM CaCl_2_). After 24 h of stabilization in the syringe, the roots were flushed three times with a clean C-free solution to remove the organic C exuded during the stabilization period. Exudate-containing samples were collected in 50 mL glass vials with silicon caps and stored at 4 °C until the determination of total organic carbon (TOC). Trap solutions containing exudates were collected from each cuvette and filtered through sterile syringe filters. A total of 32 exudate samples were analyzed (4 seedlings in two variants of moisture x four times extractions = 32). Trap solutions were then analyzed using the Shimadzu TOC—Total Organic Carbon analyzer (Shimadzu, Japan).

### Root morphology analysis

After root exudate collection, the sampled all roots were clipped from the tree, scanned at 400 dpi, and analyzed with a WinRhizo™ Pro 2003b image analysis system (Regent Instruments Inc., Ville de Québec, QC, Canada) to establish diameter, length, and projected area. Air-dried roots were further desiccated at 70 °C for 24 h to constant weight and then weighed. Root tissue density (RTD; kg m^−3^), SRA (m^2^ kg^−1^), and SRL (m g^−1^) were subsequently calculated as described by Ostonen et al.^19^. Root branching intensity was expressed as the number of root tips per 1 mg of dry mass.

### Enzyme activity analysis

The soil samples for the determination of enzymatic activity were taken from the rhizosphere zone of oak seedlings. Soil samples of natural moisture were stored at 4 °C prior to analysis. The enzymatic activity was determined at the beginning and the end of the experiment (in the 1st and 4th series of experiment). The enzyme activities were determined using fluorogenically labeled substrates^20,21^. Six fluorogenic enzyme substrates based on 4-methylumbelliferone (MUB) were used: MUB-β-D-cellobioside for β-D-cellobiosidase (CB), MUB-β-D-xylopyranoside for β-xylosidase (XYL), MUB-N-acetyl-β-D-glucosaminide for N-acetyl-β-D-glucosaminidase (NAG), MUB-β-D glucopyranoside for β-glucosidase (BG), MUB-phosphate for phosphatase (PH), and MUB-sulfate potassium salt for arylsulphatase (SP). A soil sample of 2.75 g was mixed with 92 mL buffer. After that, the soil suspension was pipetted into wells on a microwell plate containing substrate and a modified universal buffer. Fluorescence was determined by first incubating the soil suspensions for 1.5 h at 35 °C in 96-well microplates (Puregrade, Germany) and then measuring with fluorogenic substrates at an excitation wavelength of 355 nm and an emission wavelength of 460 nm.

### Statistical analysis

The Spearman correlation coefficients between different root parameters and exudation rates were calculated. Principal component analysis (PCA) was used to evaluate the relationships between root characteristics. Additionally, PCA analysis was used to evaluate the effect of moisture variant on root characteristics. The ANOVA with a Tukey test was used to assess the differences between the average values of the properties. Different lowercase letters (a, b) in tables and figures indicate significant differences in parameters between the variant of moisture. Different lowercase letters (x, y, z) indicate significant differences between the series of experiment. All the statistical analyses were performed using the statistical programs R Studio^22^ and Statistica 10.0 software (StatSoft Inc. USA 2010). The ANOVA with a Tukey test and PCA analysis were done in R while Spearman correlation was done in Statistica.

## Results

### Root carbon exudation and root morphology

**Figure 1:**
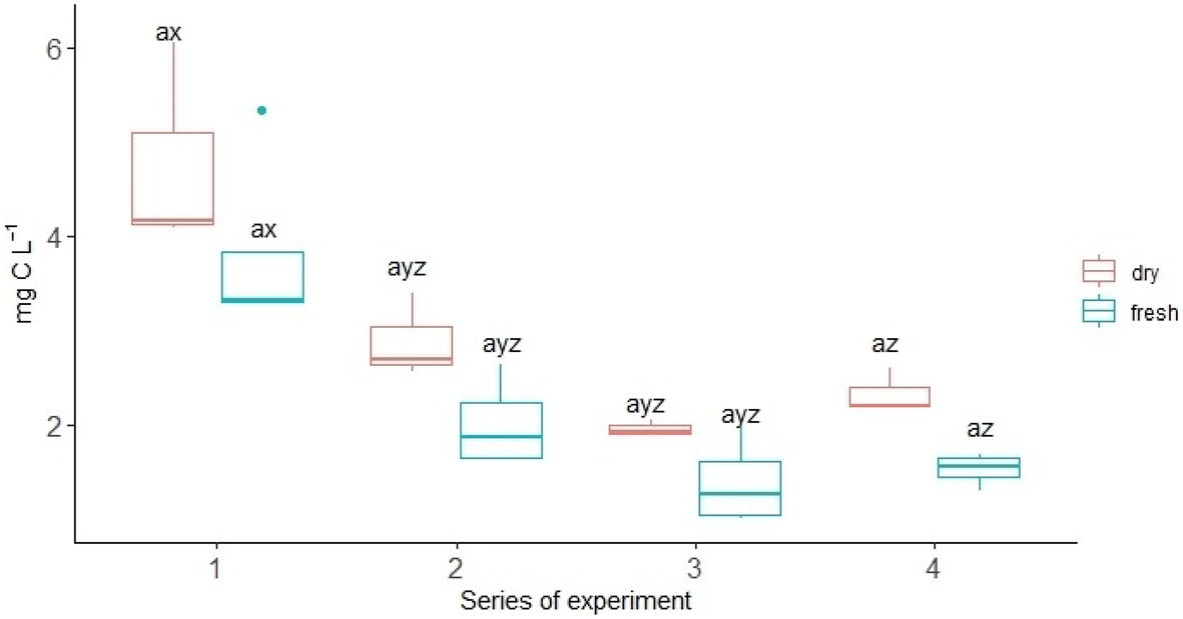
The change of total root-exuded carbon (mg C L^−1^) over time depending on the moisture variant; different lowercase letters (a,b) indicate significant differences in parameters between the different moistures; different lowercase letters (x,y,z) indicate significant differences in parameters between the series; Tukey HSD *p* < *0.05*.

**Figure 2:**
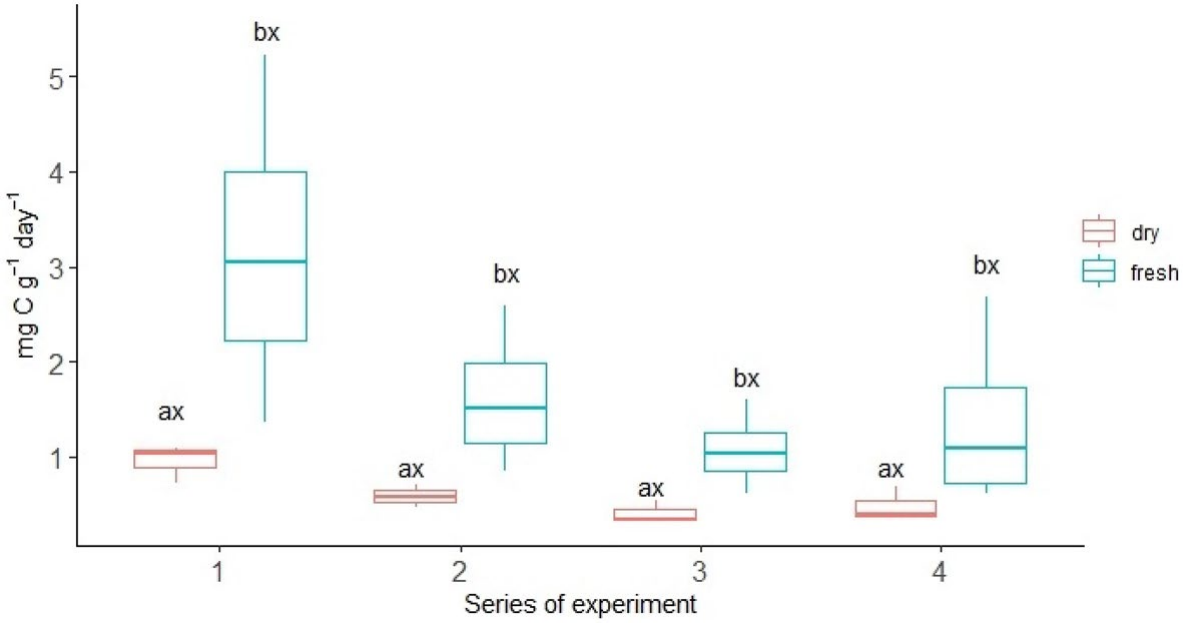
The change of root exuded C per unit root biomass over time depending on the moisture variant (mg C g^-1^ day^-1^); different lowercase letters (a,b) indicate significant differences in parameters between the different moistures; different lowercase letters (x,y,z) indicate significant differences in parameters between the series; Tukey HSD *p* < *0.05*.

On average, oak roots exuded 12.64 mg C g^−1^ day^-1^ in the fresh variant of the experiment (Table 1). The absolute amount of C root exudates collected were higher from the oak growing in the dry variant (Fig. 1). In all experimental series, there was a trend, not confirmed by statistical analysis, of a higher absolute amount of root exudate C in the dry variant compared to the fresh variant. However, when expressed per unit root biomass, this pattern disappeared. The higher root exudation rates per unit of root biomass were in the fresh variant (Fig. 2). In the first series of experiments, the root exudation rates in the fresh variant were three times higher than that of the dry variant. In subsequent series, the differences in root exudation rates in the fresh variant were smaller. In all experiment series, the root exudation rates were significantly higher in the fresh variant than in the dry variant (Fig. 2).

**Figure 3:**
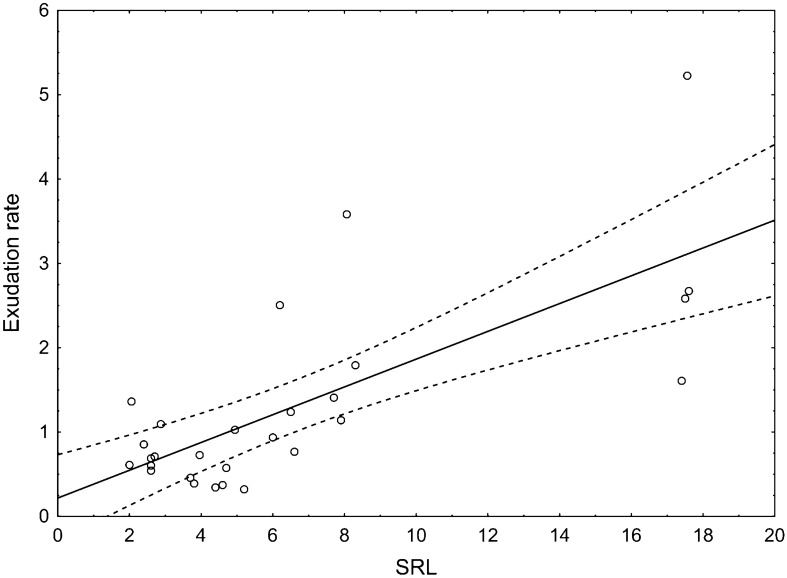
The positive relationship between root exudation rate (mg C g^-1^ day^-1^) and specific root length (SRL).

**Figure 4:**
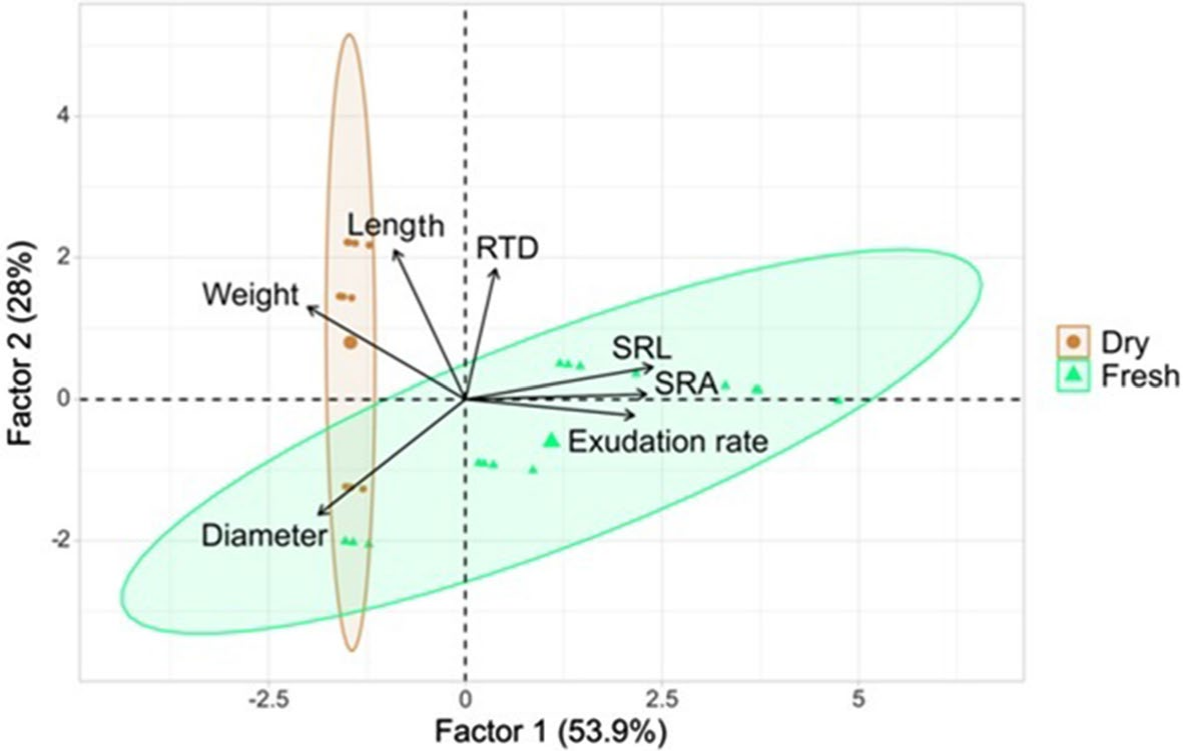
Principal component analysis (PCA) biplot of variables (root tissue density (RTD), specific root area (SRA) and specific root length (SRL), exudation rate – mg C g^-1^ day^-1^).

Moreover, the moisture variant had a significant effect on root morphology of oak (Table 2). In the case of roots in the dry variant, a higher diameter was noted. The dry variant was characterized by statistically significantly longer roots. SRA it was higher and amounted to 38.90 m^2^ kg^-1^ in the case of the fresh variant while in the case of the dry variant, it was 26.90 m^2^ kg^-1^. In the fresh variant, a higher SRL of statistical significance was noted, compared to the dry variant (8.47 m kg^-1^ and 3.92 m kg^-1^, respectively). Root tissue density did not differ between the examined variants. The exudation rate significantly correlated with the morphological features of the roots (Table 3). There was a statistically significant negative correlation between the exudation rate, diameter, and length of roots (r = -0.52 and r = -0.66, respectively). The strongest positive correlation was noted between the exudation rate and SRA (r = 0.69) and SRL (r = 0.72) (Table 3; Fig. 3). Factors 1 and 2, distinguished in the PCA analysis, explain a total of 81.9% of the variance of the tested characteristics (Fig. 4). The PCA analysis confirmed a positive correlation between the exudation rate, SRA, and SRL and a higher exudation rate in the fresh variant of the experiment.

**Table 2:** Mean morphological characteristics of roots and exudation rate (mg C g^-1^ day^-1^) in different moisture variant.

Variants	Length [cm]	Diameter [mm]	Weight [mg]	SRA [m^2^ kg^-1^]	RTD [kg m^-3^]	SRL [m kg^-1^]	Exudation rate
Fresh	65.57 ± 25.99^b^	0.20 ± 0.10^a^	10.95 ± 6.36^b^	38.90 ± 15.70^a^	64.49 ± 21.41^a^	8.47 ± 6.56^a^	12.64 ± 8.75^a^
Dry	148.13 ± 65.67^a^	0.23 ± 0.06^a^	36.43 ± 8.14^a^	26.90 ± 1.46^b^	68.11 ± 16.32^a^	3.92 ± 1.03^b^	4.23 ± 1.80^b^

Mean ± standard deviation; root tissue density (RTD), specific root area (SRA) and specific root length (SRL); a, b—statistically significant parameters; *p* < *0.05*).

**Table 3:** Correlation between different root parameters and exudation rate (mg C g^−1^ day^-1^).

	Exudation rate	Length	Diameter	Weight	RTD	SRA	SRL
Exudation rate	1.00						
Length	− 0.35	1.00					
Diameter	− 0.52*	− 0.25	1.00				
Weight	− 0.66*	0.82*	0.29	1.00			
RTD	0.09	0.29	− 0.61*	0.09	1.00		
SRA	0.69*	− 0.15	− 0.69*	− 0.63*	− 0.10	1.00	
SRL	0.72*	− 0.13	− 0.79*	− 0.61*	0.17	0.95*	1.00

*Correlation significant with *p* < *0.05.*

### Enzymes activity

**Figure 5:**
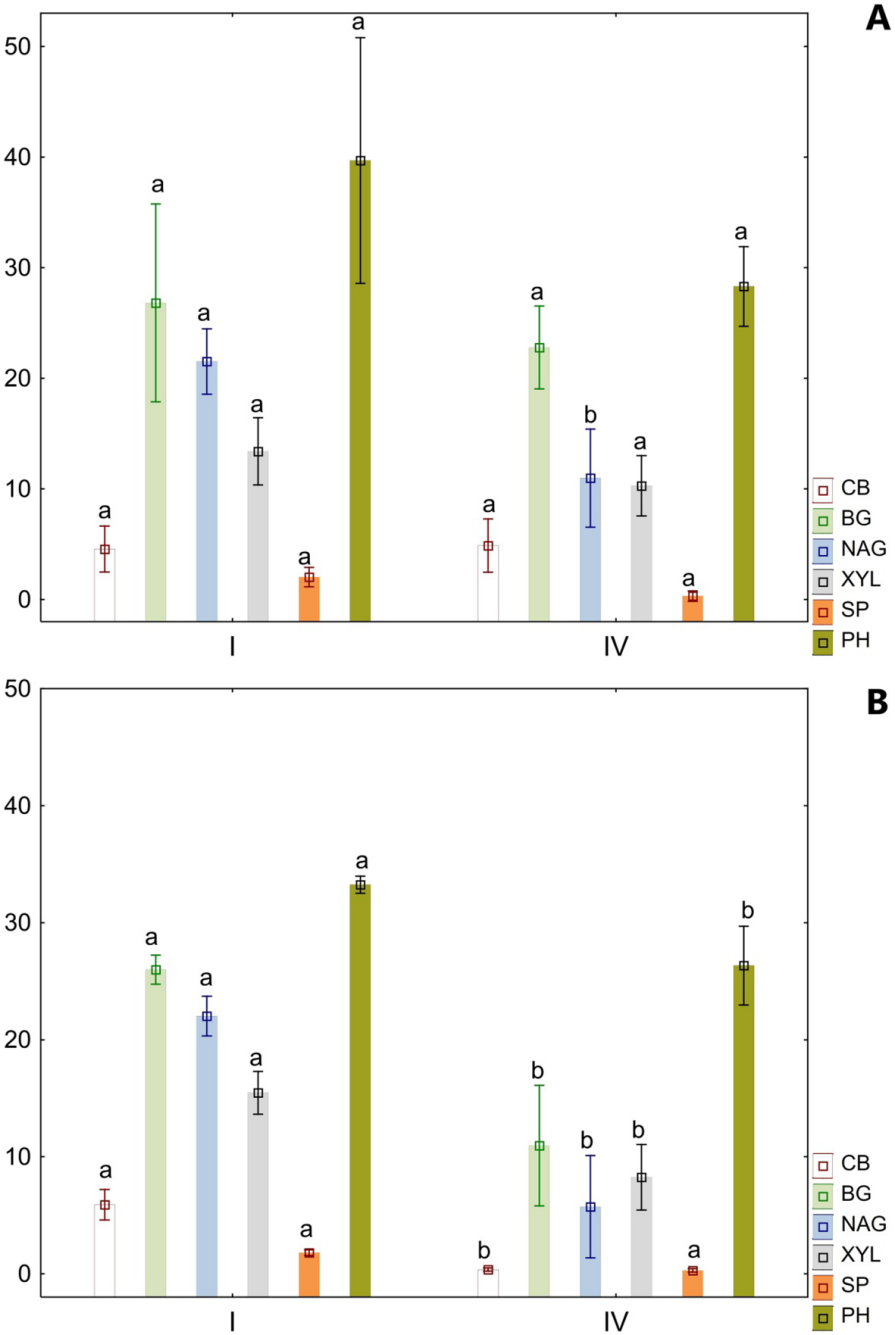
Change in enzymatic activity over time; A – fresh variant; B—dry variant; I, IV – series number; phosphatase (PH), β-glucosidase (BG), N-acetyl-β-D-glucosaminidase (NAG), β-xylosidase (XYL), β-D-cellobiosidase (CB),), arylsulphatase (SP) [nmol MUB^.^g^-1^ dry soil ^.^h^-1^]; different letters (a,b) indicate significant differences in enzyme activites between the series of experiment.

Of the tested enzymes, significantly lower activity was noted in the dry variant compared to the fresh variant (Fig. 5). In the first series of experiments, the differences in the activity of the tested enzymes were smaller. In the fourth series of experiments, when comparing the dry variant to the fresh one, enzyme activity decreased by 90% in the case of CB, 50% for BG and NAG, 20% for XYL, and 10% for both SP and PH. In the fresh variant of the experiment, significant differences between series I and IV were noted only in the case of NAG activity. However, in the dry variant, a significant decrease in the activity of all tested enzymes (except SP) was noted between series I and IV of the experiment (Fig. 5).

## Discussion

### Root morphology

This research confirmed the importance of drought in shaping the morphology of roots and root C exudation of sessile oak. The oak roots in the dry variant responded with a higher increment in length. In the case of roots growing in higher humidity, a higher SRA and SRL were determined. It is well documented that tree species adapted to dry climatic regimes generally have higher root-to-shoot ratios and deeper root systems than species from mesic climatic conditions^23^. According to Brunner et al.^24^, trees adapted to dry climates also invested more biomass into longer-lasting root organs, thus optimizing water uptake while minimizing water loss from transpiration. Such longer roots allow deeper soil penetration in search of water, typical of drought-prone plants^25^. In addition, tree species can delay drought stress by maximizing their access to water and minimizing transpirational water loss through biomass investments to the root, smaller leaf area, and strong stomatal control^26^. In this study, a decrease in soil humidity caused changes in the SRA and SRL. According to Ostonen et al.^27^. SRL is the most commonly measured morphological parameter of fine roots because it is believed to characterize the economic aspects of root systems and indicate environmental changes. Lozano et al.^28^ showed that several plant species (most forbs and some grasses) had reduced SRA and SRL as a response to drought and can be interpreted as a drought coping mechanism.

### Root exudates

Drought simultaneously affects root systems and soil nutrient availability, and roots can directly affect soil properties via the uptake of water and nutrients. Still, roots can also indirectly affect soil nutrient and C availability via root exudation^29^. This research confirmed significantly lower C exudation rates in the dry variant. The increase of the mean SRA and SRL coincided with increased C exudation. In the fresh variant, the C exudation rates were three times higher than in the dry variant. Results further show that root exudation is closely related to the morphology of the roots in drought conditions. In research by Meier et al.^9^, they show that the quantity of C released with root exudation closely and positively relates to SRL. Results in this study further confirm the role of small diameter young root segments in shaping root exudation. Sell et al.^30^ also indicated that greater mass-specific exudation flux was related to a higher proportion of pioneer roots, suggesting that an essential amount of C may be released by actively growing pioneer roots. According to Meier et al.^9^, root systems with a high SRL have, on average, thinner roots, and the C costs of root construction and mycorrhizal symbiosis are reduced. Therefore, more C may be available for root exudation.

### Enzymatic activities

The influence of drought on root growth and the intensity of root exudation induce changes in microbiological processes occurring in the rhizosphere around the roots. Karlovsky et al.^31^ analyzed the effect of simulated drought on the flux of C compounds released with exudates into the soil and their assimilation by microorganisms. They found a slowdown in microbiological processes and lower absorption of exudates by microorganisms inhabiting the rhizosphere at the culmination point of drought. In our study, the effect of reducing microbial activity was confirmed by a substantial reduction in the activity of enzymes involved in C, N, and P transformations. The most significant changes were found in the activity of CB, BG, and NAG. This decrease in enzymes activity is probably related to the availability of SOC, which is commonly the most limiting factor for microbial growth in soils. The low C supply restricted the synthesis of enzymes in the soil of the dry variant. The enhanced labile C supply from root exudation has the potential to trigger increases in decomposition rates and thus N availability^8^. The change in enzymatic activity may also be a consequence of changes in the structure and composition of microorganism species induced by the drought phenomenon. Evidence for such changes as a result of drought stress was provided by the studies of Fuchslueger et al.^32^. In the course of drought, potentially slow-growing gram-positive bacteria adapted to drought develop, and the overall ratio of fungi to bacteria changes, leading to changes in enzyme activity. Furthermore, drought induces changes in the chemical composition of exudates, as shown in the studies by Gargallo-Garrig et al.^33^, where the focus was on holm oak (*Quercus ilex*). Also, the significance of the influence of the quantity and the quality of root exudates on enzymatic activity in the rhizosphere has been confirmed in previous studies^34^. According to Zhang et al.^34^ among the tested exudate components, alanine showed the strongest stimulating effect on BG, PH, and SP activity. Hommel et al.^35^ studied the influence of drought on the rate of photosynthesis and the allocation of assimilates to the root in beech and common maple seedlings. In the case of beech, a higher assimilate allocation to the root was found under moderate drought conditions compared to an unlimited water supply. Similar experiments with sessile oak have not been carried out, but a similar phenomenon may explain the increased development of long roots found in the presented research. When considering the significant changes in the growth and structure of the root system induced by drought, namely a reduction of the degree of fine root density and a smaller number of elongation zones and root tips through which the greatest amount of exudates is subject to outflow, then the obtained results indicate a reduction in exudate excretion per unit mass of the root under the influence of simulated drought, seem to be justified.

## Conclusions

This research shows that the amount of root exudates C have a positive and close relationship with the morphology of roots, especially with SRA and SRL. Root morphology is a driver of root exudation in drought conditions. The sessile oak seedlings growing in the dry variant were characterized by lower SRA and SRL, which resulted in lower C released with root exudation. Experimental evidence has demonstrated decreased root exudation under dry conditions, which led to a change in enzyme activity. Compared to the fresh variant, enzyme activity results in the dry variant showed a decrease of 90% in CB, 50% in BG and NAG, a decrease of 20% in XYL, and SP and PH activity decreased by 10%. Knowledge about the factors shaping the accumulation of organic C in the soil and the relationship between these processes and root exudates is essential for understanding the C cycle in forest ecosystems. It is hoped that this better understanding of the mechanisms and factors influencing the dynamics of organic C in forest soils will allow for intentional prediction of these phenomena in the future, which will contribute to the prevention of the adverse effects of climate change.

## Data Availability

The data that support the findings of this study are available from the corresponding author on reasonable request.
